# The implications of varicose vein surgery on sleep evaluation scales

**DOI:** 10.3389/fpsyt.2023.1254936

**Published:** 2023-08-24

**Authors:** Ibrahim Acir, Zeynep Vildan Okudan Atay, Mehmet Atay, Vildan Yayla

**Affiliations:** ^1^Bakırköy Dr. Sadi Konuk Eğitim ve Araştırma Hastanesi, Istanbul, Türkiye; ^2^Bahçelievler Public Hospital, Istanbul, Türkiye

**Keywords:** sleep quality, varicose vein, sleep disorders, restless leg syndrome, varicose vein surgery

## Abstract

**Background:**

Varicose veins commonly occur on the legs and cause discomfort, pain, and aesthetic issues. Varicose vein surgery has an significant impact on sleep disorders such as Restless Leg Syndrome (RLS), daytime sleepiness (DS), and sleep quality (SQ). We intended to determine if preoperative and postoperative sleep quality, excessive daytime sleepiness, and RLS severity impacted in those who had varicose vein surgery.

**Materials and methods:**

The research included 160 patients who presented to the Cardiovascular Surgery outpatient clinic with symptoms of leg pain and cramping and were diagnosed with venous insufficiency. The Restless Legs Syndrome Study Group Rating Scale (RLSS), Epworth Sleepiness Scale (ESS), and Pittsburgh Sleep Quality Index (PSQI) tests were performed on patients. The patients’ scores on the scales were compared preoperatively and postoperatively.

**Results:**

The mean age of the 160 patients was calculated to be 48.7 ± 10.6 years. There were 109 female (68.1%) and 51 male (31.9%). The mean ferritin level of the patients was calculated as 61.4 mL/ng (4.3–421 mL/ng). After varicose vein surgery 63% reported improved sleep quality. Individuals with increased DS had lower postoperative RLSS scores and higher SQ. There was a decrease in postoperative RLSS scores and an increase in postoperative SQ in patients with normal DS (*p* < 0.001). Postoperative RLSS and DS scores were lower in patients with good SQ (*p* < 0.001).

**Conclusion:**

Patients had a lower RLSS score, a lower DS score, and a higher SQ after varicose vein surgery. Surgical treatment is critical to improving the quality of life and sleep comfort of patients with varicose veins and sleep disorders.

## Introduction

Varicose vein surgery is a common procedure used to treat the symptoms of varicose veins, which can cause pain, swelling, and discomfort. While the benefits of varicose vein surgery are well-documented, little attention has been paid to the impact of this surgery on sleep disorders such as Restless Leg Syndrome (RLS), daytime sleepiness (DS), and sleep quality (SQ).

We chose the Restless Legs Syndrome Study Group Rating Scale (RLSS), Epworth Sleepiness Scale (ESS), and Pittsburgh Sleep Quality Index (PSQI) as our assessment tools for specific reasons. The RLSS allows us to focus on Restless Leg Syndrome (RLS) severity, which can worsen due to varicose veins. The ESS measures daytime sleepiness, relevant to venous insufficiency’s impact. The PSQI’s comprehensive approach helps us understand how surgery affects various sleep quality aspects affected by discomfort caused by varicose veins. These established scales collectively provide a comprehensive view of how varicose vein surgery influences sleep disorders.

The ESS and PSQI are two widely used tools for assessing sleep disorders, including RLS and DS. The ESS is a self-administered questionnaire that measures a person’s level of sleepiness during the day, while the PSQI assesses the quality and quantity of sleep over a month. RLSS is used to assess the severity of restless legs syndrome, a condition characterized by an uncontrollable urge to move one’s legs.

There is some evidence that varicose vein surgery may affect sleep quality and DS. According to a study patients who underwent varicose vein treatment showed a significant increase in their sleep quality, as judged by the PSQI (1). Also it was discovered that patients reported less DS, as indicated by the ESS (2).

There is also evidence that varicose veins may worsen restless leg syndrome. According to a study, individuals who have varicose veins are more likely to report symptoms of restless leg syndrome (3).

We aimed to investigate whether preoperative and postoperative SQ, DS and the severity of restless legs syndrome evolved in individuals who had varicose vein surgery.

## Materials and methods

Patients who presented to the Cardiovascular Surgery outpatient clinic with complaints of leg pain and cramps and were considered with venous insufficiency via color Doppler ultrasonography (DUS) were included in the study. We included patients who had been diagnosed with venous insufficiency. Patients below 18 years of age were excluded from the study, as were those with a history of deep vein thrombosis. Individuals with symptomatic peripheral artery disease and a pre-existing diagnosis of restless legs syndrome were also not included. Moreover, patients who did not adhere to the post-operative follow-up appointments were excluded from the study. The radiofrequency surgery was used on 160 patients in the study. Furthermore, patients who were called for control before and after the operation (at the earliest 3 months) were evaluated using the Restless Legs Syndrome Study Group Rating Scale (RLSS), Epworth Sleepiness Scale (ESS), and Pittsburgh Sleep Quality Index (PSQI) tests. According to the RLSS scoring, 1–10 points are mild, 11–20 points are moderate, 21–30 points are severe, and 31–40 points are classified as a very severe disease. A score of 10 or higher on the ESS was associated to increased DS, while a score of 5–21 on the PSQI was associated to poor SQ.

The demographics and laboratory results of the patients were documented. The proximal saphenous vein diameter was >5.5 mm in patients with advanced vena saphenous magna reflux, according to venous Doppler ultrasonography. Patients who skipped the visit for post-operative follow-up, were under the age of 18, had a history of deep vein thrombosis, had symptomatic peripheral artery disease, or were diagnosed with restless legs were excluded from the study.

Written consent was obtained from the patients. Ethics committee approval was obtained.

### Surgery procedure

Six French sheath vena saphena magna (VSM) were placed using ultrasound at the level of the kneecap under local anesthesia using the Seldinger method. The radiofrequency method was used for VSM ablation, which was supported by tumescent anesthesia from 1 cm behind the femoral vein and VSM separation site. Then, all patients’ prominent veins were cleaned using the mini-phlebectomy method. Patients were given compression stockings and purified micronized flavonoid fraction (MPFF) in the form of 500-mg tablets twice a day for 6 months after surgery. All patients were called for follow-up appointments 1, 3, and 6 months after surgery.

### Statistically analysis

IBM SPSS Statistics for Windows 20.0 (IBM Corp., Armonk, NY, United States) was used for statistical analyses. The Shapiro–Wilk test was used to determine whether the data was normally distributed. Numerical variables with and without normal distribution were shown as mean ± standard deviation and median (min-max), respectively. Numbers and percentages were used to express categorical variables. The Student’s T test or the Mann–Whitney U test were used to assess differences between numerical variable groups. The relationship between categorical variables was tested with the Chi-Square test and Fisher’s exact test. While the statistical significance of the changes in the levels of numerical measurements after the operation was evaluated with the Wilcoxon test, and McNemar or Marginal homogeneity tests were used for categorical variables. *p* < 0.05 was considered statistically significant.

## Results

The mean age in the study, which included 160 patients, was calculated to be 48.7 ± 10.6 years. There were 109 (68.1%) female patients and 51 (31.9%) male patients. The most common symptoms among the patients were leg pain, cramps, and varicose veins, and the majority of them had a combination of them. The demographic characteristics of the patients are detailed in Table 1.

**Table 1:** Demographic and clinical characteristics [data were shown as mean ± SD or median (min-max) or numbers and percentages].

Variables	All population *n* = 160
Age, years	48.7 ± 10.6
Gender, n (%)	
Female	109 (68.1)
Male	51 (31.9)
Symptoms, n (%)	
Leg pain	152 (95.0)
Varicose vein view	125 (78.1)
Cramp	131 (81.9)
Edema	11 (6.9)
Smoker, n (%)	38 (23.8)
Preop Lab.	
Ferritin (mL/ng)	61.4 (4.3–421)
Low	22 (13.8)
Iron (μg/dL)	76 (15–201)
B12 (pg/mL)	304 (62–2000)
TSH (mU/mL)	1.6 (0.3–57.1)
T4 (ng/dL)	1.1 (0.2–4.2)
Hemoglobin (gr/dL)	13.7 ± 1.5

When examining patients’ baseline daytime sleepiness, distinctions emerge. Those with normal daytime sleepiness had a median baseline RLSS score of 20 (ranging 1–40). In contrast, patients with increased daytime sleepiness presented a higher median baseline RLSS score of 29 (ranging 17–35).

In patients with normal DS, there was a decrease in postoperative RLSS scores and an increase in postoperative SQ according to Pittsburgh. Similarly, individuals with increased DS had lower postoperative RLSS scores and higher SQ. The differences were statistically significant (*p* < 0.001) (Table 2).

**Table 2:** Change of scale measurement according to daytime sleepiness before operation [data were shown as median (min-max) or numbers and percentages].

Variables	Normal DS		*p*	Increased DS		*p*
	Preop	Postop		Preop	Postop	
RLSS	20 (1–40)	7 (1–33)	<0.001	29 (17–35)	8 (3–17)	<0.001
Mild	23 (15.5)	101 (68.2)	<0.001	0	8 (66.7)	0.002
Moderate	53 (35.8)	35 (23.6)		2 (16.7)	4 (33.3)	
Severe	59 (39.9)	9 (6.1)		6 (50.0)	0	
Very severe	13 (8.8)	3 (2.0)		4 (33.3)	0	
PSQI	5 (1–14)	4 (0–11)	<0.001	8.5 (1–12)	4 (1–10)	<0.001
Poor SQ	99 (66.9)	62 (41.9)	<0.001	11 (91.7)	5 (41.7)	<0.001

RLSS, Restless Legs Syndrome Study Group Rating Scale; ESS, Epworth Sleepiness Scale; PSQI, Pittsburgh Sleep Quality Index; DS, daytime sleepiness; SQ, sleep quality.

In patients with good SQ, there was a decrease in postoperative RLSS scores and DS (*p* < 0.001). Likewise, patients with poor SQ had lower postoperative RLSS scores and DS (*p* < 0.001). The differences were statistically significant (Table 3).

**Table 3:** Change of scale measurement according to sleep quality before operation [data were shown as median (min-max) or numbers and percentages].

Variables	Good SQ		*p*	Poor SQ		*p*
	Preop	Postop		Preop	Postop	
RLSS	19 (1–35)	4.5 (1–31)	<0.001	22 (1–40)	8 (1–33)	<0.001
Mild	10 (20.0)	40 (80.0)	0.001	13 (11.8)	69 (62.7)	<0.001
Moderate	19 (38.0)	6 (12.0)		36 (32.7)	33 (30.0)	
Severe	18 (36.0)	3 (6.0)		47 (42.7)	6 (5.5)	
Very severe	3 (6.0)	1 (2.0)		14 (12.7)	2 (1.8)	
ESS	3 (0–11)	1 (0–14)	0.004	4 (0–17)	2 (0–18)	<0.001
Increased DS	1 (2.0)	1 (2.0)	0.999	11 (10.0)	8 (7.3)	0.453

RLSS, Restless Legs Syndrome Study Group Rating Scale; ESS, Epworth Sleepiness Scale; PSQI, Pittsburgh Sleep Quality Index; DS, daytime sleepiness; SQ, sleep quality.

A decreased in RLSS scores (*p* < 0.001), a decrease in DS (*p* = 0.005), and an increase in SQ (*p* = 0.008) were noticed in both those with normal ferritin levels and those with low ferritin levels in the postoperative period, and this difference was confirmed to be statistically significant (Table 4).

**Table 4:** Change of scale measurement according to ferritin levels before operation [data were shown as median (min-max) or numbers and percentages].

Variables	Normal ferritin		*p*	Low ferritin		*p*
	Preop	Postop		Preop	Postop	
RLSS	21 (1–35)	7 (1–30)	<0.001	20 (1–40)	6 (1–33)	<0.001
Mild	20 (14.5)	94 (68.1)	<0.001	3 (13.6)	15 (68.2)	<0.001
Moderate	47 (34.1)	35 (25.4)		8 (36.4)	4 (18.2)	
Severe	62 (44.9)	9 (6.5)		3 (13.6)	0	
Very severe	9 (6.5)	0		8 (36.4)	3 (13.6)	
ESS	4 (0–16)	1 (0–15)	<0.001	4 (0–17)	3 (0–18)	0.005
Increased DS	9 (6.5)	7 (5.1)	0.727	3 (13.6)	2 (9.1)	0.990
PSQI	5 (1–14)	4 (0–11)	<0.001	6 (1–9)	5 (0–9)	0.008
Poor SQ	92 (66.7)	55 (39.9)	<0.001	18 (81.8)	12 (54.5)	0.031

RLSS, Restless Legs Syndrome Study Group Rating Scale; ESS, Epworth Sleepiness Scale; PSQI, Pittsburgh Sleep Quality Index; DS, daytime sleepiness; SQ, sleep quality.

## Discussion

The improvements in sleep quality, RLS symptoms, and DS subsequent to varicose vein surgery could be attributed to potential mechanisms including pain alleviation, mitigation of nerve irritation, enhanced blood circulation, inflammation reduction, improved psychological wellbeing, and impacts on neurological and hormonal dynamics.

Studies have shown that varicose vein surgery can improve RLS symptoms and DS (4, 5). A study conducted by Hayes et al. assessed the impact of varicose vein operation on RLS symptoms in 35 patients. The study found a significant improvement in RLS symptoms following surgery, with 80% of patients reporting an improvement in their symptoms (6). The effect of surgery on RLS and nocturnal cramps was largely investigated in these studies, but no observations on sleep quality or daytime sleepiness were recorded. In this regard, our study is more unique and comprehensive, as more sleep scales have been evaluated and objective outcomes were tried to be reached. In our research, there was a decrease in postoperative RLSS scores and an improvement in postoperative sleep quality in patients with normal daytime sleepiness. Moreover, individuals who had more DS had lower RLSS scores and higher SQ postoperative. While our study with 160 patients confirms the study conducted by Hayes et al., it also demonstrates that RLS symptoms decreased following the surgery, regardless of daytime sleepiness. The surgery has been shown to be effective in individuals with each degree of restless leg syndrome (mild, moderate, severe, or very severe). As a result, a rise in the RLSS score in varicose vein patients may be crucial in deciding the surgery.

Sleep quality got better in the first month following varicose vein surgery, according to a prospective study of 103 individuals with diabetes mellitus and chronic venous insufficiency (7). In our larger trial with more patients, individuals who had good SQ had lower postoperative RLSS score and DS. Also, those with poor SQ had lower postoperative RLS and DS feedback. In other words, the reduction in daytime sleepiness and the RLSS score in patients with good sleep quality demonstrate that the procedure has a substantial therapeutic effect on the patients’ complaints and quality of life. From this perspective, the significance of the procedure in improving the quality of life and sleep of individuals with untreated varicose veins should be considered.

Despite fewer publications claiming that ferritin levels are unrelated to RLS symptoms (8), replacement therapy is currently recommended in patients with RLS and low ferritin (9). The RLSS score was found to be significantly lower in patients with low or normal ferritin levels in the postoperative period. Therefore, regardless of ferritin level, the effect of varicose vein surgery on the RLSS score is significant. Once more, regardless of ferritin level, varicose vein surgery may be stated to decrease DS and improve SQ.

While the relationship between varicose vein surgery and sleep disorders is not yet fully understood, several possible explanations have been proposed. One possible explanation is that varicose veins can cause discomfort and pain, particularly during nighttime, leading to disrupted sleep. By removing varicose veins, patients may experience less pain and discomfort, leading to improved sleep quality. Additionally, varicose veins have been linked to RLS, which can further disrupt sleep. By removing varicose veins, patients may experience a reduction in RLS symptoms, leading to improved sleep quality.

Building on our current findings, a follow-up study could delve into the lasting effects of varicose vein surgery on sleep disorders. This extended research would provide insights into how surgery’s impact evolves over time, helping clinicians and patients make informed decisions. Tracking patients’ sleep patterns and restless leg syndrome severity beyond the immediate postoperative phase could uncover the durability of improvements. Incorporating patient-reported outcomes and quality of life measures in future studies could provide a more comprehensive assessment of varicose vein surgery’s impact on sleep. This approach would offer insights from individuals’ perspectives, capturing both subjective experiences and broader life implications. This information would guide future treatment strategies and enhance patient care. By utilizing standardized assessment tools like RLSS, ESS, and PSQI, a long-term study would ensure consistent measurement of sleep-related outcomes.

## Conclusion

Surgery led to decreased RLSS scores, indicating improved restless leg syndrome severity. Moreover, patients with normal daytime sleepiness exhibited enhanced sleep quality post-surgery, reflected by higher PSQI scores. Thus, our study emphasizes the need for post-surgery sleep disorder assessments to optimize patient care and uncover latent sleep disorders.

In conclusion, varicose vein surgery can have a positive impact on sleep disorders. Improved sleep quality can have significant benefits for patients’ overall health and wellbeing. Therefore, healthcare providers should consider assessing sleep disorders using tools such as the RLSS, ESS, and PSQI as part of their post-operative care for patients who undergo varicose vein surgery. This will ensure that patients receive optimal care and support for their recovery, and may also help to identify and treat sleep disorders that may have been previously undiagnosed.

## Data availability statement

The raw data supporting the conclusions of this article will be made available by the authors, without undue reservation.

## Ethics statement

The studies involving humans were approved by the Bakırköy Dr. Sadi Konuk Clinical Research Ethical Committee. The studies were conducted in accordance with the local legislation and institutional requirements. The participants provided their written informed consent to participate in this study.

## Author contributions

IA: Conceptualization, Investigation, Methodology, Writing – original draft. ZO: Investigation, Methodology, Project administration, Writing – review and editing. MA: Conceptualization, Methodology, Visualization, Writing – original draft. VY: Formal analysis, Supervision, Visualization, Writing – review and editing.

## Funding

The author(s) declare that no financial support was received for the research, authorship, and/or publication of this article.

## Conflict of interest

The authors declare that the research was conducted in the absence of any commercial or financial relationships that could be construed as a potential conflict of interest.

## Publisher’s note

All claims expressed in this article are solely those of the authors and do not necessarily represent those of their affiliated organizations, or those of the publisher, the editors and the reviewers. Any product that may be evaluated in this article, or claim that may be made by its manufacturer, is not guaranteed or endorsed by the publisher.
